# Time-Dependent Effects of Anesthetic Isoflurane on Reactive Oxygen Species Levels in HEK-293 Cells

**DOI:** 10.3390/brainsci4020311

**Published:** 2014-04-22

**Authors:** Yongxing Sun, Baiqi Cheng, Yuanlin Dong, Tianzuo Li, Zhongcong Xie, Yiying Zhang

**Affiliations:** 1 Geriatric Anesthesia Research Unit, Department of Anesthesia, Critical Care and Pain Medicine, Massachusetts General Hospital and Harvard Medical School, 149 13th St., 4310H, Charlestown, MA 02129, USA; E-Mails: 8793705@163.com (Y.S.); cbqharvard@gmail.com (B.C.); ydong@partners.org (Y.D.); ZXIE@mgh.harvard.edu (Z.X.); 2 Department of Anesthesia, Beijing Tongren Hospital, Capital Medical University, Beijing 100730, China; E-Mail: trmzltz@126.com

**Keywords:** isoflurane, reactive oxygen species, mitochondria

## Abstract

The inhalation anesthetic isoflurane has been reported to induce caspase activation and apoptosis, which may lead to learning and memory impairment. However, the underlying mechanisms of these effects are largely unknown. Isoflurane has been shown to induce elevation of cytosol calcium levels, accumulation of reactive oxygen species (ROS), opening of the mitochondrial permeability transition pore, reduction in mitochondria membrane potential, and release of cytochrome *c*. The time course of these effects, however, remains to be determined. Therefore, we performed a pilot study to determine the effects of treatment with isoflurane for various times on ROS levels in HEK-293 cells. The cells were treated with 2% isoflurane plus 21% O_2_ and 5% CO_2_ for 15, 30, 60, or 90 min. We then used fluorescence imaging and microplate fluorometer to detect ROS levels. We show that 2% isoflurane for 60 or 90 min, but not 15 or 30 min, induced ROS accumulation in the cells. These data illustrated that isoflurane could cause time-dependent effects on ROS levels. These findings have established a system to further determine the time course effects of isoflurane on cellular and mitochondria function. Ultimately, the studies would elucidate, at least partially, the underlying mechanisms of isoflurane-induced cellular toxicity.

## 1. Introduction

It has been reported that children who have multiple exposures (e.g., three times) to anesthesia and surgery at an early age (e.g., before age 4) may develop deficiency of cognitive function ([1,2], reviewed in [3]). In the animal studies, it has been reported that anesthesia may induce neurotoxicity and neurobehavioral deficits in rodents ([4,5,6] and monkey [7,8], reviewed in [3]). However, the cellular mechanisms of these effects remain largely to be determined.

The commonly used inhalation anesthetic isoflurane has been shown to induce caspase activation and apoptosis, and induces oligomerization and accumulation of beta-amyloid protein (Aβ) *in vitro* and *in vivo* [8,9,10,11,12,13,14,15]. Isoflurane has also been shown to induce caspase-3 activation and potentiate the nociceptive stimulation-induced cognitive impairment [16]. However, the up-stream mechanism by which isoflurane induces caspase activation and apoptosis remains largely to be determined. Reactive oxygen species (ROS) also plays an important role in Alzheimer’s disease neuropathogenesis and cognitive impairment [17,18,19,20]. Specifically, ROS may induce mitochondrial dysfunction, which then releases cytochrome *c* to the cytosol, leading to caspase-9 activation and finally caspase-3 activation (reviewed in [21]).

ROS generation is mainly through inhibition of complex I and III of mitochondria electron transport chain (ETC) [22,23]. Specifically, the electron donors NADH and FADH_2_ can be generated by the oxidation of glucose-derived pyruvate. The flow of the donated electrons (e^−^) through the ECT in the inner mitochondrial membrane pumps H^+^ ions into the inter-membrane space. ROS is generated when the voltage gradient is high because of increased flux of electron donors. However, whether the ROS generation is time dependent remains largely to be determined.

Anesthetic isoflurane has been shown to inhibit complex I of mitochondria [24], thus it is conceivable that isoflurane may increase ROS levels, via, at least partially, the inhibition of complex I. Our previous studies have shown that isoflurane can induce ROS accumulation, which may cause caspase-3 activation [25,26]. However, the time course of isoflurane’s effects on ROS is unknown. Therefore, in the present study, we set out to determine the effects of the treatment with 2% isoflurane for different periods of time (e.g., 15, 30, 60, and 90 min) on ROS levels in cultured cells. The hypothesis in the current study is that the isoflurane-induced ROS accumulation is time dependent.

## 2. Experimental Section

### 2.1. Methods

#### 2.1.1. HEK-293 Cells Culture

We used human embryonic kidney cells (HEK-293 cells) in the experiments. The cell line was cultured in Eagle’s Minimum Essential Medium (EMEM) (ATCC^®^ 30-2003™) containing 9% heat-inactivated fetal calf serum, 100 units/mL penicillin, and 100 µg/mL streptomycin. We employed HEK-293 cells in this system-generation pilot study because the HEK-293 cells with genetically-regulated mitochondrial functions are available [27], which would allow us to use both wild-type and transgenic HEK-293 cells to further assess the effects of isoflurane on ROS levels and other mitochondrial function, and furthermore, to elucidate the underlying mechanisms in the future studies.

#### 2.1.2. Treatments for Cells

Isoflurane was delivered from an anesthesia machine to a sealed plastic box in a 37 °C incubator. The box containing either 35 mm fluorodish cell culture dish (World Precision Instruments, Sarasota, FL, USA) seeded with 0.2 million cells in 1.5 mL cell culture media or 96 well plate seeded with 50,000 cells in 100 µL cell culture media. A Date infrared gas analyzer (Ohmeda, Tewksbury, MA, USA) was used to continuously monitor the delivered concentrations of carbon dioxide, oxygen, and isoflurane. The cells were treated for six hours with 2% isoflurane plus 21% O_2_ and 5% CO_2_ as described in our previous studies [25,26,28].

#### 2.1.3. ROS Accumulation Staining

OxiSelectTM ROS assay kit (Cell Biolabs, Inc., San Diego, CA, USA) was used in the experiments according to the protocol provided by the company. Briefly, 0.2 million cultured cells were placed on 35 mm fluorodish cell culture dish (World Precision Instruments) overnight in the incubator. We then added the 2,7-dichlorofluorescein diacetate (DCFH-DA)/media solution to the cells for 30 min. The DCFH-DA was rinsed by phosphate-buffered saline (PBS) twice. The DCFH-DA loaded cells were then exposed to 2% isoflurane for 15, 30, 60, and 90 min, respectively, and away from light. Finally, the cells were analyzed in warm PBS under a fluorescence microscope. Digital images were taken by a Nikon fluorescence microscopy (Nikon, Melville, NY, USA). Images were captured using 60× objective lens.

#### 2.1.4. ROS Accumulation Quantification

OxiSelectTM ROS assay kit (Cell Biolabs Inc., San Diego, CA, USA) was used in the experiments according to the protocol provided by the company. Briefly, cultured cells were placed in a clear 96-well cell culture plate overnight in the incubator. We then added the 2,7-dichlorofluorescein diacetate (DCFH-DA)/media solution to the cells. The DCFH-DA loaded cells were then exposed to 2% isoflurane for 15, 30, 60, and 90 min, respectively. The treated cells were lysed by adding 100 μL of cell lysis buffer and were mixed thoroughly and incubated for five min at room temperature. One hundred and fifty μL of the mixture was transferred to each well of a 96-well plate for fluorescence measurement. Finally, the fluorescence was read with a fluorometric plate reader (Molecular Devices, LLC, Sunnyvale, CA, USA) at 480 nm/530 nm.

#### 2.1.5. Statistical Analysis

Data were expressed as mean ± standard deviation (SD). The number of samples varied from 3 to 5. Student-*t* test was used to analyze the difference between control condition and isoflurane. *p*-values less than 0.05 were considered statistically significant. Prism 6 software (La Jolla, CA, USA) was used to analyze the data.

## 3. Results and Discussion

### 3.1. Results

**Figure 1:**
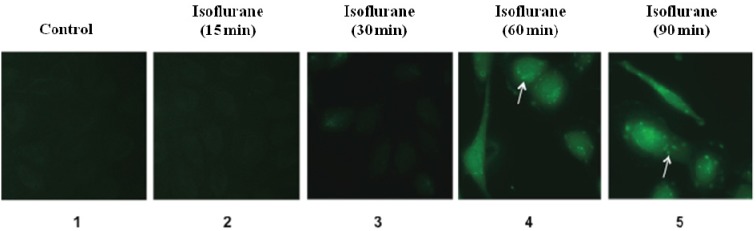
Fluorescence imaging studies of isoflurane’s effects on ROS levels in HEK 293 Cells. The green fluorescence image shows the of ROS levels in cells. (**1**) shows the ROS images in the cells treated with control condition; (**2**) shows the ROS images in the cells treated with 2% isoflurane for 15 min; (**3**) shows the ROS images in the cells treated with 2% isoflurane for 30 min; (**4**) shows the ROS image in the cells treated with 2% isoflurane for 60 min; (**5**) shows the ROS images in the cells treated with 2% isoflurane for 90 min. The treatments with 2% isoflurane for 15 and 30 min (**2**,**3**) do not induce visible increases in ROS levels inside the cells as compared to the control condition (**1**). The treatments with 2% isoflurane for 60 and 90 min (**4**,**5**) lead to accumulation of ROS inside the cells as compared to the control condition (**1**).

Our previous studies have shown that treatment with 2% isoflurane for 6 h can induce ROS accumulations *in vitro* and *in vivo* [25,26]. However, the time course of the effects of isoflurane on ROS levels remains unknown. We therefore assessed whether isoflurane was able to induce ROS accumulation with a time-dependent manner. HEK-293 cells were treated with 2% isoflurane for 15, 30, 60 and 90 min, respectively. The control condition was the carrying gas (21% O_2_ and 5% CO_2_) without isoflurane. The cells were harvested at each time point after the treatment and subjected to the fluorescence imaging. The fluorescence image showed that there was a visible increase of the fluorescence staining of ROS (green) in the cells treated with 2% isoflurane for 60 min and 90 min as compared to the control condition (Figure 1). However, the treatment with 2% isoflurane for 15 min and 30 min did not lead to visible increase in the fluorescence staining of ROS (green) in the HEK-293 cells (Figure 1). These data suggested that 2% isoflurane induce ROS accumulations only after a 60 min or longer duration of treatment. The treatment with 2% isoflurane for a short time (e.g., 15 or 30 min) did not induce ROS accumulation in the HEK-293 cells.

**Figure 2:**
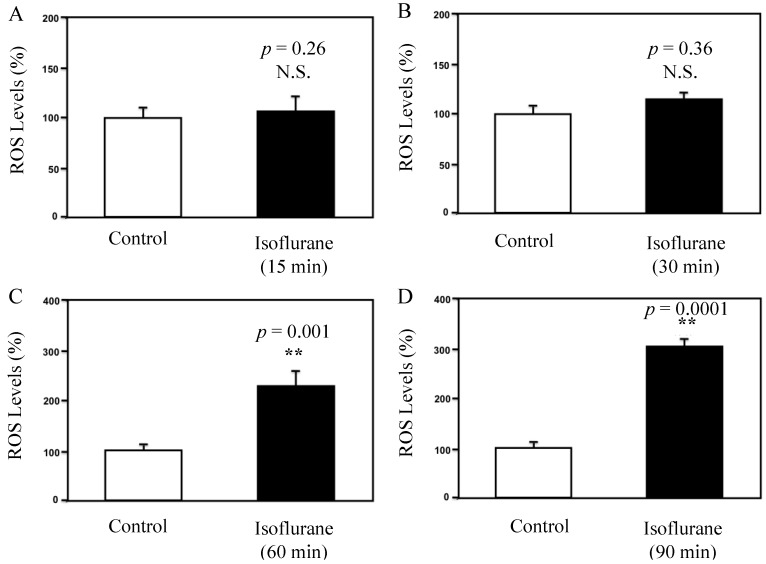
Fluorescence reading studies of isoflurane’s effects on ROS levels in HEK 293 Cells. (**A**) A fluorescence assay shows that the treatment with 2% isoflurane for 15 min (black bar) does not increase ROS levels compared with the control condition (white bar); (**B**) A fluorescence assay shows that treatment with 2% isoflurane for 30 min (black bar) does not increase ROS levels compared with the control condition (white bar); (**C**) Treatment with 2% isoflurane for 60 min (black bar) increases ROS levels compared with the control condition (white bar) (** *p* = 0.001); (**D**) Treatment with 2% isoflurane for 90 min (black bar) increases ROS levels compared with the control condition (white bar) (** p = 0.0001).

Next, we assessed the effect of isoflurane on ROS levels in the HEK-293 cells using the fluorometric plate reader. The fluorescence reading showed that the treatment with 2% isoflurane (black bar) for 15 min (Figure 2A, *p* = 0.26, N.S.) or 30 min (Figure 2B, *p* = 0.36, N.S.) did not increase the ROS levels in the HEK-293 cells as compared to control condition (white bar). However, the treatment with 2% isoflurane (black bar) for 60 min increased the ROS levels as compared to control condition (white bar) in the HEK-293 cells: 221% *vs.* 100%, ** *p* = 0.001 (Figure 2C). Finally, the treatment with 2% isoflurane for 90 min (black bar) increased the ROS levels as compared to control condition (white bar) in the HEK-293 cells: 318% *vs.* 100%, ** *p* = 0.0001 (Figure 2D).

Taken together, these data from both fluorescence image studies and fluorometric plate reader studies suggested that the isoflurane-induced ROS accumulation was time dependent. While the treatment with 2% isoflurane for 60 or 90 min increased ROS levels in the HEK-293 cells, the treatment with 2% isoflurane for 15 or 30 min did not.

### 3.2. Discussions

In current studies, we assessed the time-dependent effects of isoflurane on reactive oxygen species (ROS) accumulation in HEK-293 cells. The fluorescence imaging and fluorometric plate reader studies showed that isoflurane induced ROS accumulation in HEK-293 cells. More importantly, we found that the treatment with 2% isoflurane for 15 or 30 min did not induce ROS accumulation, but the treatment with 2% isoflurane for 60 or 90 min increased the ROS levels in the HEK-293 cells. These findings demonstrated the time-dependent effects of isoflurane on ROS levels, and showed that isoflurane might only induce ROS accumulation following a long (e.g., 60 min), but not short (e.g., 15 min), time treatment.

The findings that isoflurane can induce a time-dependent change in ROS levels will promote more studies to determine the up-stream mechanism by which isoflurane induces caspase-3 activation. Our previous studies have shown that isoflurane is able to increase calcium concentration in cytosol [29], open mitochondrial permeability transition pore, decrease mitochondrial membrane potential [30] and release cytochrome *c* [26]. Moreover, free radicals can contribute to the increased generation of ROS [31,32]. Therefore, the comparison of the time course effects of isoflurane on free radicals, ROS, cytosol, and/or mitochondrial calcium levels, mitochondrial permeability transition pore, mitochondrial membrane potential, and cytosol cytochrome *c* levels could demonstrate the potential signaling pathway underlying the isoflurane-induced caspase-3 activation.

We have found that isoflurane induced the accumulation of ROS and caspase-3 activation in H4-APP cells, B104 cells, mouse hippocampus neurons [25,26,28] in our previous studies. In the current experiments, we found that isoflurane was able to induce ROS accumulation in the HEK-293 cells. These findings have illustrated that the isoflurane-induced ROS accumulation is not cell type dependent.

ROS has been reported to have dual effects, which plays a role in both cellular protection and cellular toxicity. Specifically, ROS may contribute to the protection of heart and brain ischemia [33,34,35,36]. On the other hand, ROS has been very well shown to induce tissues damage in the heart and brain (reviewed in [37]). The dual effects of ROS could be dose-dependent [33]. The further studies may include the determination of whether different concentrations and durations of isoflurane treatments cause different amounts of ROS accumulation, leading to cellular protection or cellular toxicity. Such findings would elucidate, at least partially, the underlying mechanisms of anesthesia toxicity and anesthesia protection.

ROS generation is mainly through inhibition of complex I and III of mitochondria electron transport chain (ETC) [22,23]. Specifically, the electron donors NADH and FADH_2_ can be generated by the oxidation of glucose-derived pyruvate. The flow of the donated electrons (e^−^) through the ECT in the inner mitochondrial membrane pumps H^+^ ions into the inter-membrane space. ROS is generated when the voltage gradient is high because of increased flux of electron donors. However, whether the ROS generation is time dependent is largely to be determined.

There are several limitations in the current studies. First, we did not assess the effects of different concentrations of isoflurane on ROS levels in the studies. This was mainly because our previous studies showed that treatment with 2%, but not 1%, isoflurane for 6 hours was able to induce the caspase-3 activation in cultured cells and neurons [11,12,38]. Second, we did not compare the effects of isoflurane on other mitochondrial functions, e.g., opening of the mitochondrial permeability transition pore and mitochondrial membrane potential at different time points. However, the current findings have established a system and identified the treatment duration (e.g., 60 min) when isoflurane can induce ROS accumulation. These findings will promote a systematical investigation of the time course of isoflurane’s effects on ROS levels, mitochondrial function and cellular toxicity in our future studies. Finally, the studies were performed in human embryonic kidney cells (HEK-293 cells); therefore, the data from these studies might not be closely associated with neurotoxicity. Rather, these findings were more associated with cellular toxicity. We employed the HEK-293 cells in this system-generation pilot study to establish a system, because the HEK-293 cells stably expressed mitochondrial calcium uniporter are available [27]. In the future studies, we will compare the effects of isoflurane on ROS levels and other mitochondrial functions between the wild-type HEK-293 cells and the transgenic HEK-293 cells.

## 4. Conclusions

We have found that inhalation anesthetic isoflurane can induce the time-dependent effects on ROS accumulations in HEK-293 cells. Specifically, the treatment with 2% isoflurane for 60 or 90 min, but not 15 or 30 min, can induce ROS accumulation in cultured cells. These findings will promote more studies to investigate the effects of isoflurane on ROS levels, mitochondrial function and cellular toxicity.
